# DKA with negative DM-autoantibodies, complicated by GBS and RESLES: a case report and literature review

**DOI:** 10.3389/fimmu.2025.1597365

**Published:** 2025-09-15

**Authors:** Nan Tian, Yanwen Yang, Qiang Liu, Zeli Ma, Ying Wang, Xuan Li, Xu Wang, Yanzi Jin, Rong Chen, Yajun Li, Fenkui She, Fenglin Mou, Qing Zhang

**Affiliations:** ^1^ Clinical Medical College, Ningxia Medical University, Yinchuan Ningxia, China; ^2^ Department of Neurology, General Hospital of Ningxia Medical University, Ningxia, Key Laboratory of Cerebrocranial Disease, Incubation Base of National Key Laboratory, Yinchuan, Ningxia, China; ^3^ Department of Neuroelectrophysiology, Cardiovascular and Cerebrovascular Disease Hospital Branch, General Hospital of Ningxia, Yinchuan, Ningxia, China

**Keywords:** GBS, DM, DKA, RESLES, autoantibody-negative

## Abstract

Guillain-Barré syndrome (GBS) is an autoimmune-mediated disorder characterized by acute, multifocal peripheral neuropathies, typically triggered by infections, autoimmune diseases, or immunosuppressive therapies. In this report, we describe a patient newly diagnosed with diabetes mellitus (DM) and diabetic ketoacidosis (DKA), who presented with negative insulin-related autoantibodies and no identifiable infectious etiology. It is suggested that DKA may independently contribute to the development of GBS. Additionally, DKA can lead to the reversible splenial lesion syndrome (RESLES). We present a case of GBS with atypical features consistent with the acute motor axonal neuropathy (AMAN) variant.

## 1 Introduction

DKA is a life-threatening acute complication of DM characterized by hyperglycemia, metabolic acidosis and ketonemia. It most commonly occurs in individuals with type 1 diabetes and is typically associated with the presence of diabetes-related autoantibodies, including glutamic acid decarboxylase antibody (GAD-Ab), insulinoma-associated protein 2 antibody (IA-2A), and insulin autoantibodies (IAA). However, a subset of patients presenting with DKA may lack detectable levels of these autoantibodies, thereby complicating the accurate classification and diagnosis of diabetes subtypes.

In rare cases, DKA may be complicated by neurological disorders, underscoring the intricate interplay between metabolic disturbances and neurological dysfunction. In this case, a young woman experienced delayed diagnosis of DM, presenting with DKA accompanied by coma and respiratory distress. Upon regaining consciousness, physical examination revealed signs of peripheral neurological deficits. Cerebrospinal fluid (CSF) analysis demonstrated albuminocytologic dissociation, and electromyography indicated symmetrical axonal and demyelinating lesions, leading to a diagnosis of GBS. During hospitalization, brain magnetic resonance MRI and DWI revealed findings consistent with RESLES. Follow-up imaging confirmed complete resolution of the lesions, establishing the diagnosis of RESLES.

This case highlights the diagnostic and therapeutic challenges posed by the coexistence of these rare conditions. It aims to explore potential pathophysiological links between DKA, GBS, and RESLES, and to provide a comprehensive review of the relevant literature. We hope this report will contribute to a deeper understanding of these uncommon associations and support clinicians in the timely recognition and management of similar complex cases.

## 2 Case presentation

**Figure 1:**
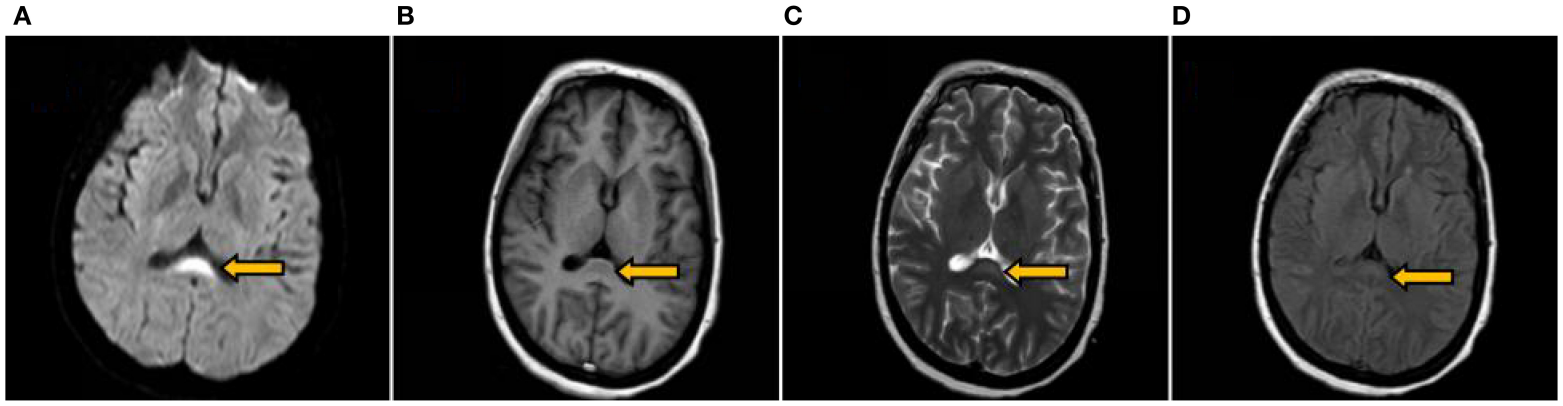
Brain MRI on the 9th day after symptom onset. Revealing an oval symmetrical lesion (indicated by the arrow) located in the central region of the splenium of the corpus callosum. The lesion appeared hyperintense on DWI **(A)** hypointense on T1WI **(B)** and exhibited slight hyperintensity on both T2WI **(C)** and T2Flair **(D)**.

A 37-year-old woman was admitted with a 10-day history of paroxysmal dizziness and confusion, which had worsened over the preceding 4 days. On June 17, 2024, she developed dizziness, nausea, and fatigue after consuming chilled watermelon, without accompanying headache, vomiting, or vertigo. She was initially diagnosed with acute gastroenteritis at a local clinic and treated with intravenous glucose, saline, and oral antacids. However, she lapsed into a coma approximately two hours after treatment. On June 18, 2024, she was transferred to a county hospital, where laboratory investigations confirmed a diagnosis of DKA. She received insulin therapy, intravenous fluid resuscitation, and empirical measures to prevent infection. By June 21, the patient regained consciousness but presented with dysarthria and restricted limb movement. On June 23, she developed dyspnea and respiratory distress, requiring endotracheal intubation and mechanical ventilation. During hospitalization, she experienced recurrent episodes of fever, coma, nausea, and vomiting. Neurological examination revealed bilateral fixed mydriasis (6.0 mm) and absence of pupillary light reflexes. On June 26, the patient was transferred to our hospital. Brain MRI, including DWI, demonstrated abnormal signal intensities in the splenium of the corpus callosum (**Figure 1**). The initial diagnosis remained DKA. Notably, the patient had no prior history of diabetes, and both her personal and family medical histories were unremarkable.

Admission: The patient exhibited a body temperature of 37.8°C, a respiratory rate of 21 breaths per minute, a pulse of 134 beats per minute, and a blood pressure of 82/43 mmHg. Endotracheal intubation was performed, and mechanical ventilation was initiated to provide respiratory support. Pulmonary auscultation revealed diminished breath sounds and moist rales bilaterally in the lower lung fields. Cardiac examination showed a heart rate of 134 beats per minute with a regular rhythm and no audible murmurs or abnormal heart sounds. The abdominal examination was unremarkable. The patient was sedated and receiving analgesics. Mild limb withdrawal was observed in response to light orbital pressure. Pupils were bilaterally equal, round, and fixed at 6.0 mm, with no reaction to light. Corneal reflexes were preserved bilaterally, while the oculocephalic reflex was absent on both sides. There was symmetrical flattening of the forehead and nasolabial folds. Muscle tone was globally reduced without evidence of muscle atrophy. Deep tendon reflexes were absent in all four limbs, and no pathological reflexes were elicited. A complete neurological assessment could not be performed due to lack of patient cooperation.

Local county hospital tests: Blood glucose 32.7 mmol/L, urine glucose 4+, ketone bodies 3+, blood gas analysis showed pH 6.798, lactate 1.48 mmol/L, base excess (B) -32.7 mmol/L; electrolyte values included Na^+^ 150 mmol/L, Cl^-^ 109.6 mmol/L, K^+^ 1.83 mmol/L. Glycosylated hemoglobin was 14.00% (reference range 3.6-6%). Respiratory and viral tests for respiratory syncytial virus, influenza A and B, adenovirus, human rhinovirus, and Mycoplasma pneumoniae were negative. Blood culture showed no pathogenic bacterial growth after five days of incubation.

Tests in our hospital: cerebral pressure was 172mmH2O, and CSF analysis showed a white blood cell count of 5/mm³, CSF protein 7.22 g/L (reference 0.12-0.60 g/L), and CSF IgG 519.00 mg/L (reference 0 – 34 mg/L). Blood tests showed Na+ 148.6 mmol/L, Cl- 115.5 mmol/L, K + 4.49 mmol/L, lactate 1.40 mmol/L, and blood glucose 9.7 mmol/L (with insulin pump). Ketone bodies were positive initially but negative after 2 days. Insulin autoantibodies were negative. White blood cell count was 11.55×10^9^/L with 69.4% neutrophils. Preoperative tests for Hepatitis B, syphilis, Hepatitis C, and HIV were negative. Routine Electrocardiogram (ECG) showed sinus tachycardia (heart rate 105 bpm). Electromyography (EMG) indicated severe motor nerve involvement in both upper and lower extremities, with abnormal peripheral motor conduction in bilateral facial nerves, showing axonal and demyelinating damage. Brain MRI (1.5T, plain and diffusion-weighted) revealed abnormal signals in the splenium of the corpus callosum (**Figure 1**). Chest HRCT identified bilateral pneumonia with minimal right pleural effusion and bilateral pleural thickening.

Diagnosis: 1. Diabetes, 2. DKA, 3. GBS, 4. RESLES, 5. Pulmonary infection (bilateral).

**Figure 2:**
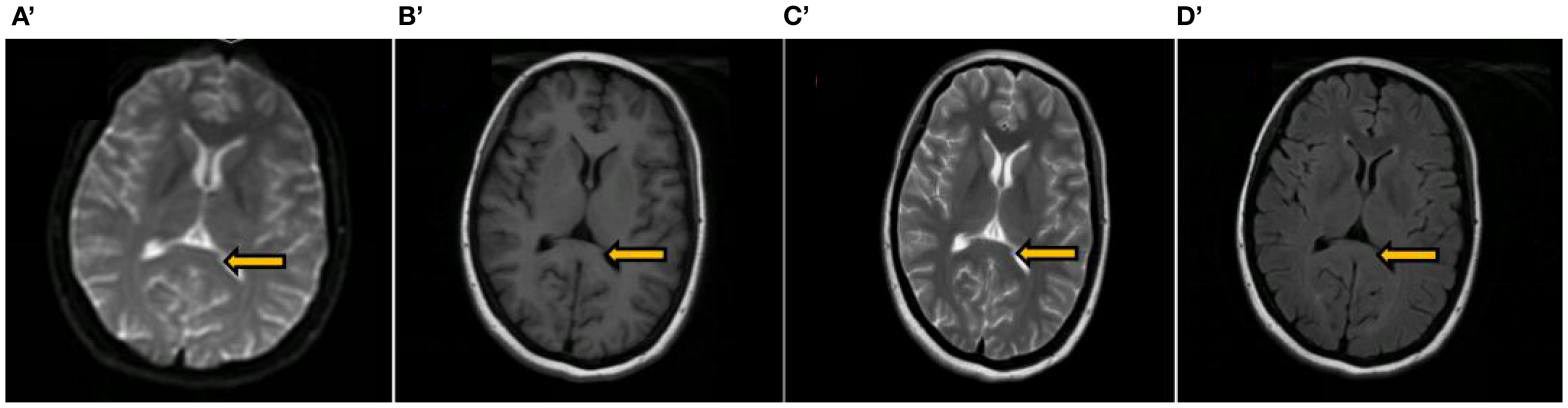
A review of the brain MRI conducted 2 months post-discharge. The images include the splenium of the corpus callosum (indicated by an arrow), along with DWI image **(A’)** T1-weighted image **(B’)** T2-weighted image **(C’)** and T2 Flair image **(D’)** all of which displayed no abnormal signals.

Treatment and Process: Upon admission, the patient was diagnosed with DKA and treated with mechanical ventilation, gradual hypoglycemic management, extensive fluid infusion, infection prevention, B vitamins, and rehabilitation. Blood glucose was stabilized. Tachycardia (100–140 bpm) and intermittent hypotension (75-110/40–75 mmHg) persisted, treated symptomatically. On the fourth day after admission, lumbar puncture was performed, and CSF analysis revealed albuminocytologic dissociation. Combined with the electromyographic findings of predominantly axonal damage in multiple, symmetric motor nerves, a definitive diagnosis of GBS of the AMAN subtype was established. The patient received intravenous immunoglobulin (IVIG) 0.4 g/kg/h. Nine days post-admission, physical examination revealed consciousness, absent cough reflex, no bilateral light reflex, bilateral ocular movements(including adduction, abduction, elevation, and depression)were completely restricted, corneal reflexes were symmetrically present on both sides, the oculocephalic reflex was absent bilaterally, bilateral facial paralysis, and weakened pharyngeal reflexes. Muscle strength: grade 4 in upper limbs, grade 3 in proximal lower limbs, grade 2+ in distal lower limbs, muscle tone in all four limbs was decreased, with no sensory deficits observed in the face, trunk, or extremities. Heart rate and blood pressure stabilized. After 32 days, the patient was weaned off the ventilator and swallowing improved. On discharge, facial paralysis persisted with slightly shallow nasolabial folds, but the patient could smile. One month later, peripheral facial paralysis and muscle strength normalized, and brain MRI showed resolution of the abnormal signal in the corpus callosum splenium (**Figure 2**).

## 3 Discussion

The young woman with no prior history of DM presented with a blood glucose level of 32.7 mmol/L, ketone bodies at 3+, pH 6.798, lactate 1.48 mmol/L, and a base excess of -32.7 mmol/L, along with a glycosylated hemoglobin of 14.00%, leading to a diagnosis of DM and DKA. Clinically, she exhibited symmetrical limb weakness, cranial nerve palsies (III, IV, VI, VII, IX, X, XI, XII), and respiratory distress, suggestive of phrenic and spinal nerve involvement. Additionally, persistent tachycardia, intermittent hypotension, and pupil dilation pointed to autonomic dysfunction. CSF analysis showed protein-cell dissociation, while EMG revealed a symmetric, predominantly axonal polyradiculoneuropathy affecting multiple motor nerves, supporting a diagnosis of GBS. Brain MRI and diffusion-weighted imaging suggested RESLES, with follow-up imaging confirming resolution of the splenium abnormality.

GBS is an autoimmune-mediated disorder characterized by multiple peripheral neuropathies, often triggered by infections (particularly Campylobacter jejuni), autoimmune conditions, or immunosuppressive therapy. In GBS, the immune system targets peripheral nerve components resembling pathogens, leading to nerve damage. Autonomic dysfunction, such as blood pressure and heart rate variations, occurs in approximately two-thirds of GBS cases and is typically transient, which aligns with this patient’s presentation (1). No DM-related autoantibodies were detected, suggesting a low likelihood of secondary autoimmune disease. DKA may independently trigger GBS, as supported by similar case reports (2, 3). Potential mechanisms include: (1) DKA’s association with infection, which could trigger an immune response against nerve tissue, though no specific infection was identified in this case; (2) acute polyneuropathy, primarily affecting motor nerves, occurring during DKA treatment, with rapid glucose correction possibly contributing (4). In this case, insulin was administered at a moderate rate (0.1U/kg/h), with no high-dose infusions, reducing the likelihood of insulin-related neuropathy. Additionally, DKA-induced acidification from elevated ketone bodies could disrupt normal protein function (5), potentially leading to structural and functional neuronal abnormalities that trigger GBS.

To date, no reports have linked DKA to RESLES. However, based on current research into the pathogenesis of RESLES, it is hypothesized that DKA may contribute to electrolyte imbalances, oxygen demand-supply discrepancies, hyperglycemia, hyperosmolality, rapid fluid infusion, and swift hypoglycemic responses, all of which could disrupt fluid and electrolyte distribution, leading to cytotoxic edema in the splenium of the corpus callosum. This region connects the two cerebral hemispheres and consists mainly of white matter fiber tracts. The vascular density in this region is low and its vascular regulation mechanism may be different from that of gray matter, which makes the vasculature prone to imbalance in regulation leading to edema (6). Anneken (7)used DTI to show that the cytotoxic edema in RESLES is confined to glial cells and myelin sheaths, without damaging the nerve fibers themselves. Glial cells and myelin sheaths in the splenium uptake glutamate, leading to cellular edema while preventing energy failure in nerve cells, which may explain the reversibility of the edema (8, 9). Clinically, RESLES presents with nonspecific symptoms resembling encephalitis or encephalopathy (10), and focal neurological deficits may be obscured due to altered consciousness from sedation or analgesia used during mechanical ventilation.

In summary, delayed intervention in this case allowed DM to progress to DKA, which was secondary to GBS and RESLES. While DKA can trigger GBS, the symptoms of ketosis-induced coma may obscure clinical signs of GBS, increasing the risk of missed diagnosis. This is especially critical in severe GBS cases, where delayed immunotherapy can negatively impact outcomes. If unexplained limb weakness, cranial nerve involvement, and dependence on mechanical ventilation arise after conventional treatment, particularly when sensory deficits are mild, GBS should be strongly suspected. Early EMG, CSF analysis, and other diagnostic tests are essential to avoid missed diagnoses and ensure timely treatment. The nonspecific clinical features of RESLES make early craniocerebral MRI and imaging studies crucial to reduce diagnostic and treatment delays. Treatment focuses on addressing the underlying condition, with prompt diagnosis and management often leading to a favorable prognosis (11). This case underscores that DKA is a rare but significant trigger for both GBS and RESLES, highlighting the need for further research into its pathogenesis, diagnosis, and treatment to improve patient prognosis and quality of life.

## Data Availability

The original contributions presented in the study are included in the article/**Supplementary Material**. Further inquiries can be directed to the corresponding author.
