# Supplementary material for: DKA with negative DM-autoantibodies, complicated by GBS and RESLES: a case report and literature review
Source: Front Immunol. 2025 Sep 15;16:1597365. doi: 10.3389/fimmu.2025.1597365 (PMC12477141; doi:10.3389/fimmu.2025.1597365)

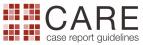

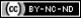

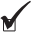
**CARE Checklist of information to include when writing a case report**


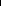


**Topic**

**Title**

**Key Words**

**Abstract**

**(no references)**

**Introduction**

**Patient Information**

**Clinical Findings Timeline**

**Diagnostic Assessment**

**Therapeutic Intervention**

**Follow-up and Outcomes**

**Discussion**

**Patient Perspective Informed Consent**


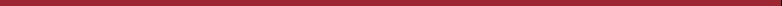


**Item Checklist item description Reported on Line**

**1** The diagnosis or intervention of primary focus followed by the words “case report” . . . . . . . . . . . . . . . . . . . . . . . Line 1-2 / Page 1

**2** 2 to 5 key words that identify diagnoses or interventions in this case report, including "case report" . Line 21 / Page 1 **3a** Introduction: What is unique about this case and what does it add to the scientific literature? . . . . . . . . . . . . . . Line 22-42 / Page 1-2 **3b** Main symptoms and/or important clinical findings . . . . . . . . . . . . . . . . . . . . . . . . . . . . . . . . . . . . . . . . . . . . . . . . . . . . Line 44-72 / Page 2 **3c** The main diagnoses, therapeutic interventions, and outcomes . . . . . . . . . . . . . . . . . . . . . . . . . . . . . . . . . . . . . . . . . Line 100-119 / Page 3-4 **3d** Conclusion—What is the main “take-away” lesson(s) from this case? . . . . . . . . . . . . . . . . . . . . . . . . . . . . . . . . . . . Line 162-174 / Page 5

**4** One or two paragraphs summarizing why this case is unique (**may include references**) . . . . . . . . . .Line 23-42 / Page 1-2 **5a** De-identified patient specific information. . . . . . . . . . . . . . . . . . . . . . . . . . . . . . . . . . . . . . . . . . . . . . . . . . YES **5b** Primary concerns and symptoms of the patient. . . . . . . . . . . . . . . . . . . . . . . . . . . . . . . . . . . . . . . . . . . . . . . . . . .Line 44-72 / Page 2 **5c** Medical, family, and psycho-social history including relevant genetic information . . . . . . . . . . . . . . . Line 58-59 / Page 2 **5d** Relevant past interventions with outcomes . . . . . . . . . . . . . . . . . . . . . . . . . . . . . . . . . . . . . . . . . . . . . .Line 44-59 101-119-70 / Page 2 3-4

**6** Describe significant physical examination (PE) and important clinical findings. . . . . . . . . . . . . .Line 60-72 109-118 / Page 2 3-4

**7** Historical and current information from this episode of care organized as a timeline . . . . . . . Line 44-59 101-119 / Page 2 3-4 **8a** Diagnostic testing (such as PE, laboratory testing, imaging, surveys). . . . . . . . . . . . . . . . . . . . . . . . . . . . .Line 60-119 / Page 2-4 **8b** Diagnostic challenges (such as access to testing, financial, or cultural) . . . . . . . . . . . . . . . . . . . . . . . . . . . NA

**8c** Diagnosis (including other diagnoses considered) . . . . . . . . . . . . . . . . . . . . . . . . . . . . . . . . . . . . . . . . . . . . . . . .Line 100/ Page 3 **8d** Prognosis (such as staging in oncology) where applicable . . . . . . . . . . . . . . . . . . . . . . . . . . . . . . . . . . . . . . . . Line 117-119 / Page **9a** Types of therapeutic intervention (such as pharmacologic, surgical, preventive, self-care) . . . . . . . . . . . . . . . . . . .Line 101-119 / Page 3-4 **9b** Administration of therapeutic intervention (such as dosage, strength, duration) . . . . . . . . . . . . . . . . . . . . . . . . . . . . Line 101-119 / Page 3-4 **9c** Changes in therapeutic intervention (with rationale) . . . . . . . . . . . . . . . . . . . . . . . . . . . . . . . . . . . . . . . . . . . . . . . . . . . Line 101-119 / Page 3-4

**10a** Clinician and patient-assessed outcomes (if available) . . . . . . . . . . . . . . . . . . . . . . .. . . . . . . . . . . . . . . . . . . . . . . . . .Satisfied **10b** Important follow-up diagnostic and other test results . . . . . . . . . . . . . . . . . . . . . . . . . . . . . . . . . . . . . . . . . . . . . . . . . . Line 118-119 / Page 4 **10c** Intervention adherence and tolerability (How was this assessed?) . . . . . . . . . . . . . . . . . . . . . . . . . . . . . . . . . . . . . . .Satisfied **10d** Adverse and unanticipated events . . . . . . . . . . . . . . . . . . . . . . . . . . . . . . . . . . . . . . . . . . . . . . . . . . . . . . . . . . . . . . . . .NA **11a** A scientific discussion of the strengths AND limitations associated with this case report . . . . . . . . . . . . . . . . . . . . .Line 38-42 / Page 1-2 **11b** Discussion of the relevant medical literature **with references**. . . . . . . . . . . . . . . . . . . . . . . . . . . . . . . . . . . . . . . . Line 131-174 / Page 4-5 **11c** The scientific rationale for any conclusions (including assessment of possible causes) . . . . . . . . . . . . . . . . . . . . . .Line 131-174 / Page 4-5 **11d** The primary “take-away” lessons of this case report (without references) in a one paragraph conclusion . . . . . Line 162-172 / Page 5


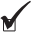
**12** The patient should share their perspective in one to two paragraphs on the treatment(s) they received . . . . . .Satisfied

**13** Did the patient give informed consent? Please provide if requested . . . . . . . . . . . . . . . . . . . . . . . . . . . . . . . . . . . . . . **Yes**
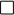
 **No**
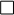

Supplement: Supplementary file 1 [file Table1.docx]
